# Development of an Ultrasound Technique to Evaluate the Popliteal Complex in the Horse

**DOI:** 10.3390/ani12070800

**Published:** 2022-03-22

**Authors:** Merete Møller-Jensen, Michaela Hansen Blomquist, Camilla Lamhauge Mortensen, Isolde Katharina Christersdotter Olsson, Gabriel Cuevas-Ramos

**Affiliations:** Large Animal Teaching Hospital, Department of Medicine and Surgery, University of Copenhagen, 1165 Copenhagen, Denmark; meretemj1992@gmail.com (M.M.-J.); michaela.blomquist@yahoo.dk (M.H.B.); cpm304@alumni.ku.dk (C.L.M.); isolde.katharina.olsson@gmail.com (I.K.C.O.)

**Keywords:** ultrasound, popliteal tendon, popliteal muscle, equine, stifle

## Abstract

**Simple Summary:**

The popliteal tendon and muscle are major stabilizers of the human and dog knee. Injury to this complex causes knee pain, and it is generally associated with other injured structures such as the lateral meniscus and/or the cranial cruciate ligament. The equine popliteal complex is poorly reported. Lameness due to stifle pathology is a serious clinical concern in sport horses, and the roll of the popliteal complex in this is unknown. One of the cardinal diagnostic tools on lameness exams is ultrasonography; however, a comprehensive technique to examine the complete popliteal complex (tendon and muscle) in horses has not been reported. The objective of the study was to develop a detailed ultrasound technique of the equine popliteal complex. We present here a detailed ultrasound technique to clearly evaluate the popliteal tendon, its components and variable insertions, the subpopliteal recess, and muscle. This new ultrasound approach is easy to apply by following clear anatomical landmarks, even by inexperienced operators. The technique presented here can be complementary to a routine stifle ultrasound exam.

**Abstract:**

The popliteal tendon and muscle are major stabilizers of the human and dog knee, more specifically the postero-lateral corner. Injury to this complex causes posterior knee pain, and it is generally associated with other injured structures such as the lateral collateral ligament, lateral meniscus, and/or the cranial cruciate ligament. The popliteal complex is poorly reported in the horse, and its specific function has not been determined. Nevertheless, it is likely that it is similar to that of other species, and that injury to it could have similar clinical repercussions. Lameness due to stifle pathology is a serious clinical entity in sport horses. One of the cardinal diagnostic tools in lameness exams is ultrasonography; however, a comprehensive technique to examine the popliteal complex (tendon and muscle) in the horse has not been reported. The objective of the study was to develop a systematic ultrasound technique of the equine popliteal complex that allows identification of the insertion and variations of the popliteal tendon (PopT), as well as examination of the popliteal muscle (PopM). Comparison between anatomical variants among horses was studied in order to identify possible significant differences. For this, 10 university teaching horses were used, hence 20 stifles were examined, several times. With the ultrasound technique presented here, the PopT and PopM are consistently examined. The developed technique allows reliable examination of the popliteal complex in the horse, and it could be included during standard ultrasound examination of equine stifle.

## 1. Introduction

There is scarce literature regarding the function of the equine popliteal complex (tendon and muscle), and descriptions of how to examine these anatomical structures are limited. The lack of knowledge potentially causes the popliteal complex to be overlooked during lameness and imagery examinations, thus the clinical impact cannot be determined when potential injury has occurred. 

In both humans and canine, the popliteal tendon (PopT) is considered a stabilizing structure of the posterior-lateral region of the knee, principally during flexion [1,2]; thus, a similar function in the horse could be assumed, with the associated clinical repercussions. The mechanism of injury to this specific area is considered to involve hyperextension and varus force of high energy [2,3]. In humans, injury of the PopT typically occurs in association with other structures, such as the cruciate ligaments, lateral meniscus, and lateral collateral ligament (LCL) [4]. In humans, injury to the popliteal muscle (PopM) is considered more common than injury to the tendon alone. A study with 24 patients with injury to the popliteal complex, showed that 95.8% involved the muscular portion and associated injuries occurred in 91.7% (22/24) of patients [4]. In horses, stifle injuries often have important clinical repercussions [5,6], and it is therefore possible that similar pathological changes and affections of the popliteal complex may occur, where the muscle could be more often affected, as it is the case in humans. 

**Figure 1:**
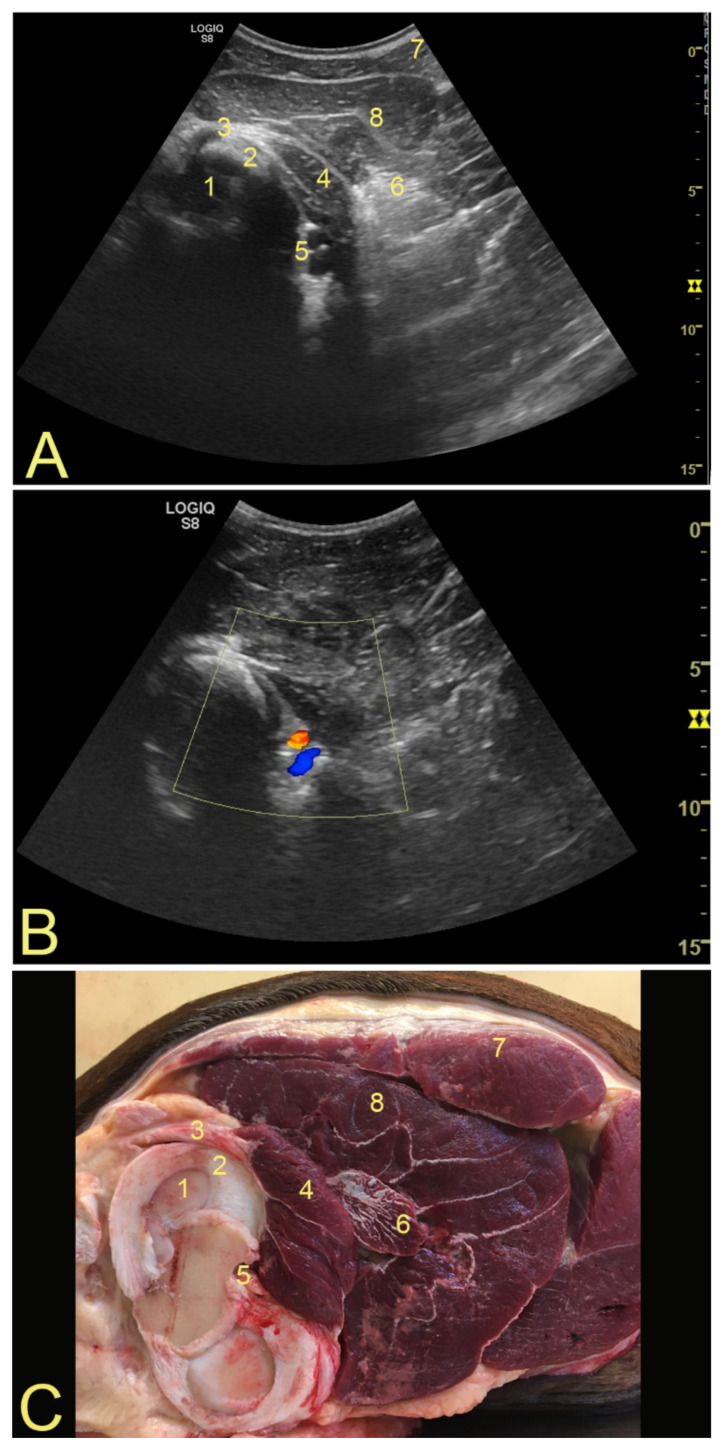
(**A**) Ultrasound aspect of the popliteal muscle. (**B**) Normal aspect and anatomy of the popliteal muscle and surrounding structures. To confirm the correct position of the probe, a doppler mode was used to demarcate the popliteal artery and vein. (**C**) An anatomical specimen is shown for orientation. Annotations: 1—caudo-lateral part of the lateral femoral condyle; 2—lateral meniscus; 3—PopT; 4—PopM; 5—popliteal artery and vein; 6—fibrous superficial digital flexor muscle; 7—biceps femoralis muscle; 8—gastrocnemius muscle.

Anatomically, the PopM runs from the proximo-lateral tibia, just caudal to the stifle, to the medial third of the tibia, where it inserts along its medial border. It is surrounded by the popliteal artery and vein, the fibrous superficial digital flexor muscle, and the gastrocnemius muscle (Figure 1C). The popliteal tendon inserts over the lateral femoral epicondyle, it is intrasynovial, and similar to humans, the equine PopT has three variants (types I, II, and III) depending on the number of components forming the tendon, it can be conformed from one to three parts. In contrast to humans where these components can be separated, in the horse they are stacked together, separated by visible septa [7]. Additionally, in the lateral epicondyle of the femur, the localization of the tendon insertion can vary in regard to the LCL [7]. This means that taking as a point of reference the LCL, the PopT insertion can be found cranial, underneath, or caudal to it. In this same study [7], it was reported that in 66.7% of examined stifles, the PopT insertion is located cranial to the LCL, in 25% caudal to it, and in 8.3% the tendon inserted underneath the LCL. These findings, although not the same, could be compared to what is found on human anatomy [1]. We could not find in the literature however, on both humans and horses, an association between these anatomical variations and a stronger or weaker chance of injury. Nonetheless, what has been reported in humans is that pathology of the popliteal complex is linked to clinical signs of knee instability and pain in the lateral and posterior regions [8]. In the horse, there are only two reported clinical cases, one associated with an avulsion fragment at the site if insertion of the PopT [9], and the other one linked to a traumatic event provoking a popliteal tendonitis [10]. Both cases were related to other injured tissues within the lateral femoro-tibial joint (LFTJ), as it is often described in human reports. However, there are no reports, in the equine literature, on pathology found within the PopM or the musculo-tendinous region of this complex. 

Ultrasonography is one of the gold standard diagnostic modalities in musculoskeletal injuries in horses, and has been shown to be a cardinal tool for diagnosis of soft tissue injuries within the equine stifle. A systematic ultrasound approach of the stifle has been described [11]. Despite the intrinsic ultrasound limitations, it has shown to be effective in terms of visualizing the menisci, patellar ligaments, collateral ligaments, cranial meniscal ligaments, and a portion of the cranial cruciate ligament [12]. Even if the technique to ultrasound the PopT on its own exists, a detailed approach on how to ultrasound the equine popliteal complex (tendon and muscle) and its variants (tendon type and site of insertion) has not been reported. The aim of the study was to develop a detailed ultrasound technique of the equine popliteal complex that will allow identification of the PopT and its anatomical variations, and allow examination of the PopM, its ultrasonographic appearance and localization. Comparison between anatomical variants and among horses was studied in order to identify possible significant differences.

## 2. Materials and Methods

Ten university teaching horses were used, 20 stifles were examined by three examiners independently, so to develop and validate the ultrasound technique. Animals were included following guidelines of animal welfare in agreement with the local ethical committee. Noninvasive, nor painful procedures were done in these animals. Ultrasound examinations took place with the animal standing, without sedation. Lateral and caudal regions of both stifles were clipped and prepared for image acquisition. 

For anatomical purposes, a cadaver specimen stifle was obtained from an animal euthanized for reasons unrelated to this study. The same anatomical landmarks used for the ultrasound technique were used to identify the saw transection point, and used of the cut stifle for anatomical illustration.

A LOGIQ S8 (LOGIQ^TM^) ultrasound machine (General Electric Company GE, Buckinghamshire, UK) was used, PopT images were obtained with a linear probe with a frequency of 7.5 Hz, and images of the PopM were obtained with a convex probe with a frequency of 3 Hz. Images were stored and analyzed with a PACS system. Anatomical landmarks, depth for image acquisition, and limb and probe positioning were first established. Three different examiners obtained images independently (MHB, CLM, and GCR). The type of PopT was established with a transverse view of the tendon within the subpopliteal recess, and the insertion variant was determined in accordance with the location of the LCL at the lateral femoral epicondyle level [7]. The tendon can insert either cranial, underneath, or caudal to the LCL, and the tendon can be conformed of one, two, or three parts [7].

Statistical analysis was performed with either Excel or Graph-Pad Prism7 software (GraphPad Software, San Diego, CA, USA). Normal distribution, T-test, and regression tests were used. The aim was to identify possible significant variations between observers, among animals, type of tendon insertion, muscle and tendon width, and their combinations. The tendon width was measured on longitudinal ultrasound images. The muscle width was determined by a line starting at the popliteal artery and vein, and ending at the border of the fibrous superficial digital flexor muscle (Figure 1C). Statistical analysis was done to compare possible associations between tendon width and type of insertion, muscle width versus tendon insertion, height of the horse, left or right limbs. Significance was set at *p* < 0.05. The objective was to identify any possible differences that could be related to the different normal anatomical variations that are found in the equine popliteal complex.

## 3. Results

### 3.1. Anatomical Landmarks for Probe Positioning and Technical Settings

During the examination, the horse was maintained in a standing position fully weight bearing and with squared hind limbs. To evaluate the PopT, a linear ultrasound probe was first placed over the lateral meniscus, caudal to the extensorius sulcus, with a depth of 6 cm, in a longitudinal position (proximal to distal). Once the meniscus was located, the linear probe was displaced caudally until the LCL was found and centered on the screen. Then the probe was rotated about 30 to 45 degrees—anticlockwise if you are assessing the left hindlimb, or clockwise if you are assessing the right hindlimb (Supplementary Materials Video S1)—in order to localize the PopT and obtain a longitudinal view of it. Depending on the location of the insertion of the PopT in relation to the LCL, the probe had sometimes to be displaced caudally or cranially (Supplementary Materials Video S1). Once a longitudinal image of the PopT was obtained, the probe was rotated 90 degrees in situ so a transverse view of the tendon could be obtained, which also allowed evaluation of the subpopliteal recess. Following these steps, the PopT was consistently imaged. In a longitudinal image, the tendon has a linear fibrillar pattern that is similar to other tendinous structures, with an homogeneous echogenicity. In transverse views, the tendon has a regular architecture with a shape that is somehow triangular to rectangular.

To evaluate the PopM, and because it is a deeper and wider structure, a convex probe was used, with a 15 cm depth image. By placing a finger at the location of the meniscus, the convex probe was placed in a transverse position (cranial to caudal), just cranial to the edge of the biceps femoralis muscle, at the level of the lateral meniscus (Supplementary Materials Figure S1). The convex probe was directed cranio-proximal, until the caudal edge of the lateral femoral condyle could be visualized. The PopT could then be seen running longitudinally which helped to recognize the PopM. The muscle was located between the fibrous part of the superficial digital flexor muscle and the popliteal artery and vein (Figure 1) (Supplementary Materials Video S2).

### 3.2. Popliteal Complex Characteristics

**Figure 2:**
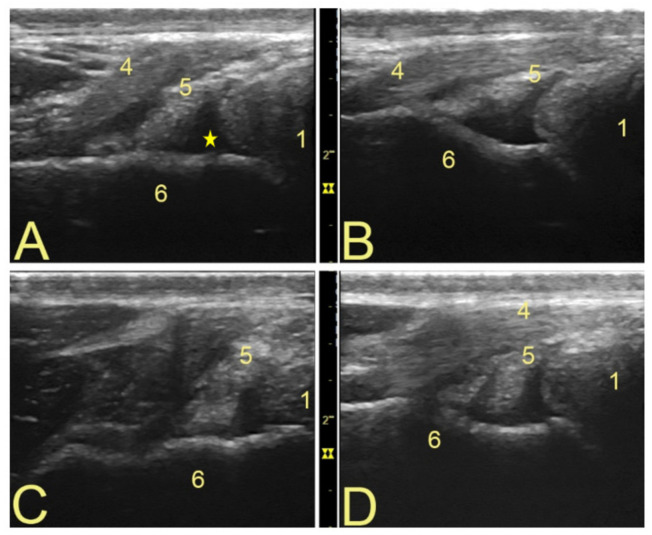
Variants on popliteal tendon structure and aspect of the subpopliteal recess. The variants on tendon components were identified with a transverse view. This view also shows hypoechogenic synovial fluid within the subpopliteal recess. Cranio-proximal is to the left. (**A**–**D**) Different tendon shapes and components (7). The LCL also changes in aspect according to the placement of the probe, as it changes according to the insertion type of the PopT. Annotations: 1—lateral meniscus; 4—LCL; 5—PopT; 6—lateral femoral epicondyle, yellow star marks synovial fluid.

**Figure 3:**
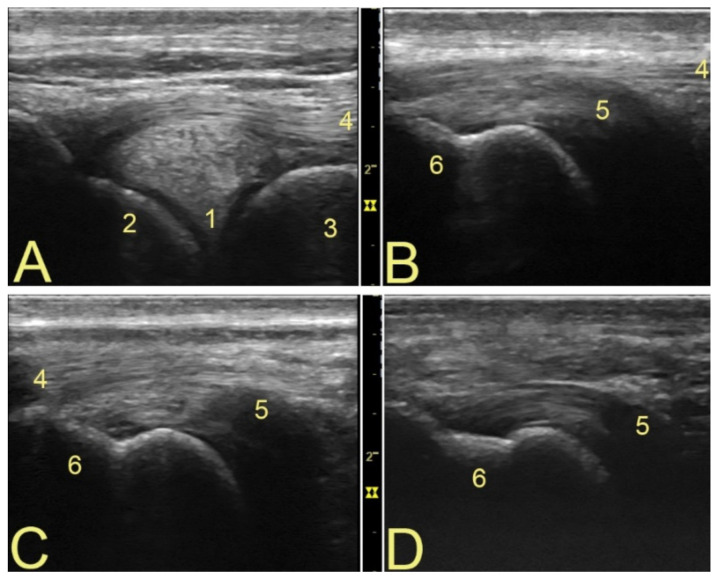
Insertion variants of the popliteal tendon. The type of insertion of the PopT depends to its relation to the LCL [7]. Proximal to the left. (**A**) Lateral meniscus and LCL. (**B**) Cranial insertion of the PopT, the LCL cannot be fully visualized when the full width of the tendon is visible. (**C**) When the insertion of the PopT is located underneath the LCL, the latter structure is clearly seen in the image. (**D**) Caudal insertion of the PopT, as the probe had to be displaced caudally (Supplementary Materials Video S1), the LCL is no longer visible while the full width of the tendon is. Annotations: 1—lateral meniscus; 2—lateral femoral condyle; 3—tibia; 4 LCL; 5—PopT; 6—lateral femoral epicondyle.

There was no significant variation between observers’ measurements and image annotations (confidence level 95%). Among the ten horses examined, 60% of PoPT insertions were localized cranial to the LCL, 30% caudal to it, and 10% underneath. The mean width of the PopT was 0.49 cm ± 0.14 cm, and that of the PopM was 2.11 cm ± 0.49 cm. The subpopliteal recess had discrete amounts of hypoechogenic fluid in all transverse images, but it was hard to identify it longitudinally. No synovial villi could be seen. Variations on the components of the tendon [7] could be evaluated only on the transverse view (Figure 2). Longitudinally, the architecture of the tendon fibers and the structure of the femoral epicondyle were easier to evaluate, together with the location of insertion of the tendon in relation to the LCL [7] (Figure 3). The muscle bundle is relatively small, with very stable anatomical landmarks. No significant differences in muscle size or architecture could be detected between animals (Figure 1).

**Figure 4:**
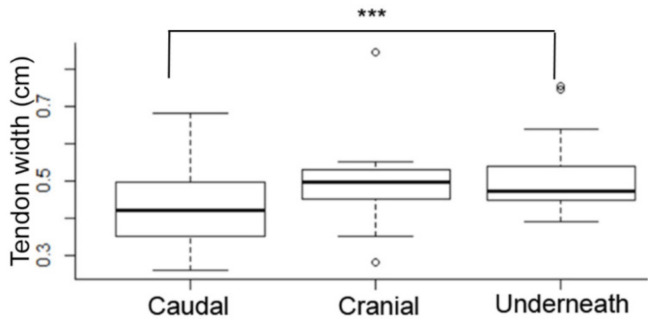
Tendon width versus tendon insertion type ANOVA analysis. When comparing the width of the tendon with the insertion type there was a significance difference only between the width of the caudal and underneath insertions. Meaning that the tendon with a caudal insertion is smaller than the tendon with an underneath insertion type. Cranial insertion versus caudal insertion had a *p-*value of 0.2 (not significant). Underneath insertion versus caudal insertion had a *p* 0.001 (***). Cranial insertion versus underneath insertion had a *p* 0.57 (not significant). The *x*-axis shows the type of tendon insertion in relation to the LCL.

**Figure 5:**
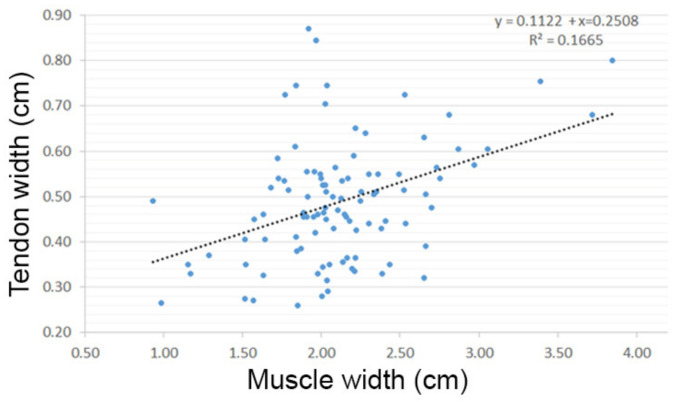
Regression analysis on tendon width versus muscle width. Analysis showed a R-value of 0.408, R^2^ of 0.16, and a *p-*value of 0.0002. It is expected then that a higher muscle width will correlate with a higher tendon width.

Statistical analysis showed that when comparing the width of the tendon with the insertion type there was a significant difference between the width of the caudal and underneath insertions (*p* = 0.001). Meaning that the tendon with a caudal insertion is smaller than the tendon with the underneath insertion type (Figure 4). Additionally, there was a significant correlation (R value of 0.408, R^2^ of 0.16) between tendon width and muscle width (*p* = 0.0002), indicating that the thicker the tendon is, the thicker the muscle is (Figure 5). When testing the height of the horse against the muscle and tendon width, there was significant difference showing that a taller horse had a significantly thicker tendon (*p* = 0.01) and muscle (*p* = 0.001). Comparing the width of the muscle with the tendinous insertion type, there was a significant difference between the width of the muscle when a tendon was inserted caudally versus underneath the LCL (*p* = 0.0001), and between the cranial versus underneath types (*p* = 0.028). This indicates that the muscle is larger when the horse has a tendon insertion underneath the LCL.

## 4. Discussion

This newly developed ultrasound technique can clearly identify the popliteal complex along the caudo-lateral aspect of the equine stifle. It can be performed with ease, and by following anatomical landmarks that are describe above, the technique was straightforward to apply even by unexperienced operators. Furthermore, the technique can identify the different tendon types and insertional variants [7]. The tendon can be conformed of one to three parts that are separated by a septum of fascia-like tissue. The point of insertion of the PopT can change in regard of the position of the LCL, as it can be situated cranial to the LCL, underneath it, or caudally [7] (Figure 2 and Figure 3). The PopM was successfully examined, no variations in terms of form, position, or size, were observed.

The anatomical course of the PopT has been described recently as a landmark for arthroscopy of the caudal compartments of the LFTJ [13], the tendon is shown running deep to the LCL in a caudal to distal direction, while enclosed within the subpopliteal recess. The insertion site of the PopT was not discussed, but changes in tendon conformation were also observed during arthroscopy. However, as the location of the PopT insertion can vary, it can potentially modify the entry point to the caudal compartments of the stifle, not meaning that this will not allow arthroscopic evaluation, but that it could modify the landmarks used, or the camera point of observation. Ultrasound examination following the technique described in the present study could help determine the localization of the PopT insertion, and possibly prevent confusion when approaching the caudal compartments of the LFTJ via the subpopliteal recess.

On lameness localized to the stifle, it is currently unknown which repercussions could develop if the PopM becomes atrophied, due to abnormal use of the limb for instance, as it happens with the gluteal muscles or the multifidus muscle [14], this study provides information about the average size and echographic appearance of this muscle. The PopM is an important stabilizer of the knee, and it has been reported that 95.8% of lesions to the human popliteal complex involved the muscle and/or the musculotendinous part [4,15], causing postero-lateral knee instability and pain. Additionally it is unknown if a caudal insertion of the PopT is somehow weaker, as statisticly, we show that it represents the thinner tendon in terms of width. In horses, chronic hind limb lameness could lead to PopM damage or atrophy, and possibly subsequent stifle instability, pain, and further lameness. Stifle injuries are of great clinical importance in the equine athlete [5,6]. In humans, damaged to the PopM has been shown to increase the risk of osteoarthritis of the medial femoro-tibial joint (MFTJ) [16]. This could be of relevance in equine medicine as osteoarthritic changes within the MFTJ have long been recognized as a cause of lameness [17], unfortunately there are no reports in horses evaluating the popliteal complex when the MFTJ is affected.

There are no reports describing strain tendonitis of the equine PopT, only traumatic lesions have been reported in two occasions [9,10]. Thus, the true prevalence of tendonitis affecting the popliteal complex is unknown, as these structures are not systematically examined. In contrast to humans, the equine PopT is intra-articular, it is surrounded by synovium, forming what has been referred to as the subpopliteal recess [7,13], and can be easily examined with the ultrasound technique presented in this study. This is of importance because whenever tendonitis would occur, it would likely have repercussions within the LFTJ. Moreover, in humans, injury to the PopT is generally associated with trauma to the LCL or lateral meniscus [4], hence injury to these structures in the horse, could mean potential pathology of the PopT as well. However, in the horse, the popliteal complex is generally overlooked. This study provides reference values in terms of tendon width, muscle size, and the different correlations between structures.

The popliteal complex plays an important role as knee stabilizer in both humans and dogs. Pathology within its structures can affect not only the LFTJ but also the MFTJ, and it is often associated with trauma to the LCL and lateral meniscus. Even if unknown, it is possible that similar pathogenesis would occur in the horse. Therefore, lameness localized to the stifle should include examination of this complex. 

Limitations of this study are the small population size, and the use of only non-lame horses. 

## 5. Conclusions

This study shows an ultrasound method to examine the popliteal complex (tendon and muscle) in the horse. The anatomical course of the PopT and PopM were visualized, and the anatomical variants were confirmed. The technique can easily be applied even if examiners are unexperienced.

## Data Availability

Additional ultrasound images and statistical analysis files are available if required.

## Supplementary Materials

The following supporting information can be downloaded at: https://www.mdpi.com/article/10.3390/ani12070800/s1, Figure S1: Probe placement for popliteal muscle scanning; Video S1: Probe placement to scan the caudal popliteal tendon insertion. Video S2: Popliteal artery and vein.
