# Supplementary figures and images for: Development of an Ultrasound Technique to Evaluate the Popliteal Complex in the Horse
Source: Animals (Basel). 2022 Mar 22;12(7):800. doi: 10.3390/ani12070800 (PMC8996960; doi:10.3390/ani12070800)

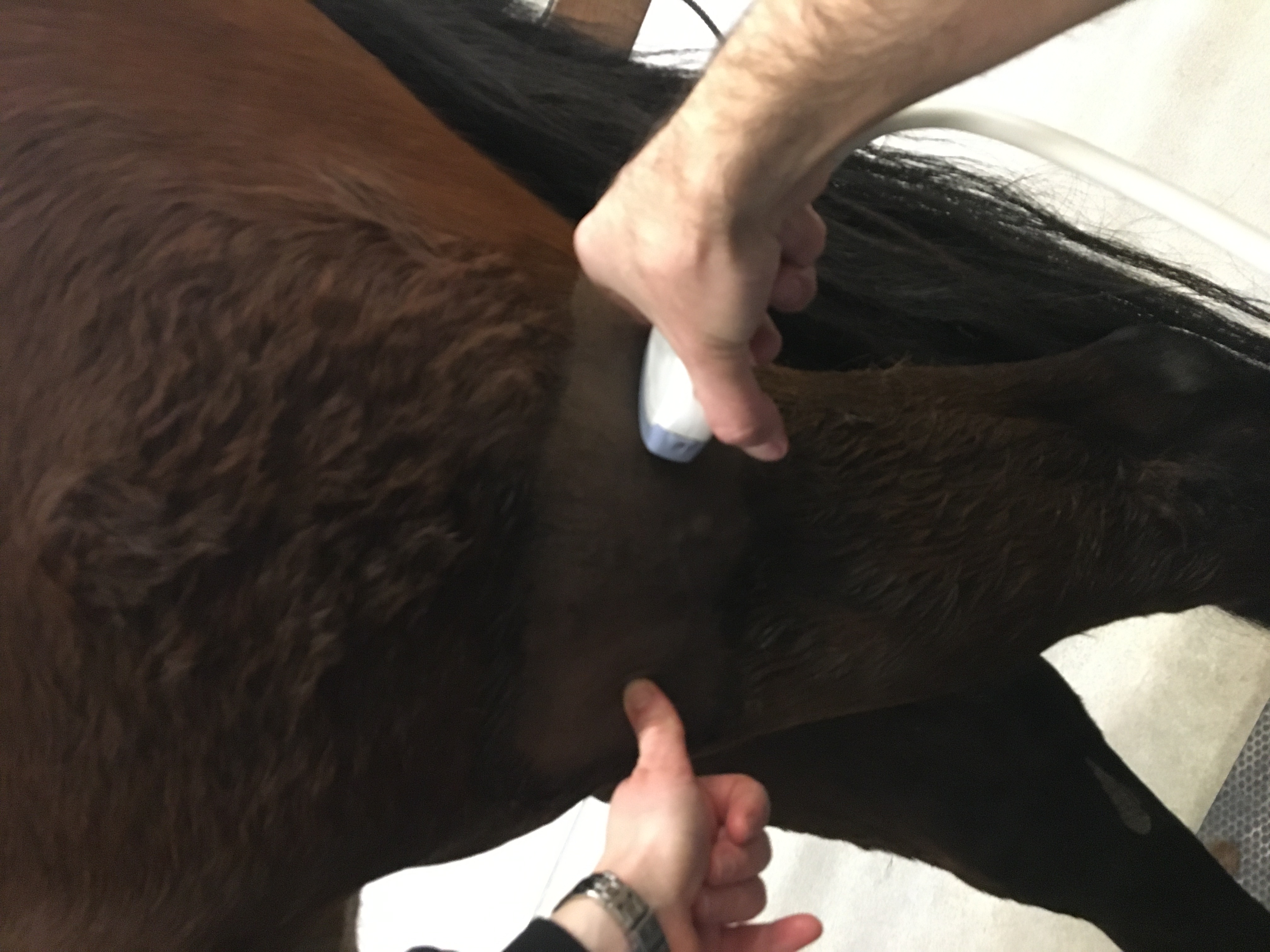

Supplement: Supplementary file 1 [file animals-12-00800-s001.zip › Suppl Figure S1.JPG]
